# A 20-gene mutation signature predicts the efficacy of immune checkpoint inhibitor therapy in advanced non-small cell lung cancer patients

**DOI:** 10.1186/s12890-023-02512-6

**Published:** 2023-06-22

**Authors:** Xilin Hu, Jing Guo, Jianguang Shi, Da Li, Xinjian Li, Weijun Zhao

**Affiliations:** Department of Thoracic Surgery, The First Affiliated Hospital of Ningbo University, 315010 Ningbo, China

**Keywords:** NSCLC, ICI, Predictive biomarker, Tumor mutational burden, Gene mutation signature, Nomogram

## Abstract

**Background:**

There is an unmet need to identify novel predictive biomarkers that enable more accurate identification of individuals who can benefit from immune checkpoint inhibitor (ICI) therapy. The US FDA recently approved tumor mutational burden (TMB) score of ≥ 10 mut/Mb as a threshold for pembrolizumab treatment of solid tumors. Our study aimed to test the hypothesis that specific gene mutation signature may predict the efficacy of ICI therapy more precisely than high TMB (≥ 10).

**Methods:**

We selected 20 candidate genes that may predict for the efficacy of ICI therapy by the analysis of data from a published cohort of 350 advanced non-small cell lung cancer (NSCLC) patients. Then, we compared the influences of various gene mutation signatures on the efficacy of ICI treatment. They were also compared with PD-L1 and TMB. The Kaplan-Meier method was utilized to evaluate the prognosis univariates, while selected univariates were adopted to develop a systematic nomogram.

**Results:**

A high mutation signature, where three or more of the 20 selected genes were mutated, was associated with the significant benefits of ICI therapy. Specifically, patients with high mutation signature were confirmed to have better prognosis for ICI treatment, compared with those with wild type (the median PFS: 7.17 vs. 2.90 months, p = 0.0004, HR = 0.47 (95% [CI]:0.32–0.68); the median OS: unreached vs. 9 months, p = 1.8E-8, HR = 0.17 (95% [CI]:0.11–0.25)). Moreover, those patients with the high mutation signature achieved significant ICI treatment benefits, while there was no difference of OS and PFS between patients without the signature but TMB-H (≥ 10) and those without the signature and low TMB(< 10). Finally, we constructed a novel nomogram to evaluate the efficacy of ICI therapy.

**Conclusion:**

A high mutational signature with 3 or more of the 20-gene panel could provide more accurate predictions for the outcomes of ICI therapy than TMB ≥ 10 in NSCLC patients.

**Supplementary Information:**

The online version contains supplementary material available at 10.1186/s12890-023-02512-6.

## Introduction

A breakthrough in cancer treatment over the past decade has been the advent of immune checkpoint inhibitor (ICI). In single-agent or combination therapy, ICI have provided robust and durable responses in a subset of advanced non-small cell lung cancer (NSCLC) patients, dramatically extending overall survival (OS) [1–3]. However, only a small portion of potential candidates respond to ICI treatment [4]. There is an unmet need to identify biomarkers that enable more accurate identification of individuals who can benefit, due to the high cost involved with ICI treatments and probable severe adverse effects in some treated patients [5]. Several biomarkers have been identified to be associated with the sensitivity of ICI therapy, including high programmed death-ligand 1 (PD-L1) expression [2, 6, 7], a high tumor mutational burden (TMB) [6, 7], microsatellite instability-high (MSI-H) [8] and a deficiency in mismatch repair (MMR)[9].

As the most widely used biomarker for immunotherapy, PD-L1 expression in tumor cells, is frequently employed in pembrolizumab treatment for NSCLC in both first-line and second-line [10, 11]. However, nivolumab does not extend progression-free survival (PFS) in patients with high PD-L1 levels when used in the first line setting [12]. As a result, ICI efficacy has not yet been reliably predicted by PD-L1 level. MSI (Microsatellite instability) is also a reliable genetic indicator of tumor responsiveness to ICI therapy [9, 13, 14]. A small percentage of malignancies have microsatellite instability because their mismatch repair genes are defective. Malignancies with MSI + are associated with better response [13, 14]. MSI + status appears to generate more tumor-specific neoantigens and increases potential for immune system recognition [15–17]. Another appropriate predictive biomarker for immunotherapy efficacy is TMB [18–20]. Multiple reports demonstrate that the effect of a higher mutational burden is consistent with the idea that neoantigens can promote immune system recognition [21]. However, it is difficult for TMB as a biomarker for ICI treatment because TMB differs across tumor types [22] and various types of cancers appear to have different cut-offs [17]. The US FDA recently approves TMB score of ≥ 10 mut/Mb as a threshold for pembrolizumab treatment of solid tumors [23] but the suitability of TMB-H needs to be proved by further clinical researches.

The specific somatic mutation in driver genes could promote tumor cell growth and affect the prognosis of ICI treatment. Patients who had EGFR or ALK mutations may not have therapeutic improvements from ICIs [24, 25]. Co-occurring genomic alterations of TP53 and LKB1 play a key role in altering the tumor microenvironment of NSCLC and are associated with the susceptibility to ICI therapy [26]. Moreover, the mutation of ARID1, FGFR4, and ERBB4 may serve as a new biomarker for the sensitivity to ICI treatment and prognosis of advanced NSCLC [15, 27, 28]. This study examined the hypothesis that compared to TMB status, the efficacy of ICI therapy might be predicted more accurately by particular gene mutations that were correlated with a survival benefit of ICI treatment. In the end, a systematic nomogram based on clinicopathological information and mutational data was developed to predict the prognosis of NSCLC patients receiving ICI therapy.

## Methods

### Source of patient treatment and mutation data

Clinical and mutational data of immunotherapy cohorts were from Memorial Sloan-Kettering Cancer Center (MSKCC) and obtained from the cBioPortal database (https://www.cbioportal.org). Two separate cohorts were utilized in our study, one for selecting mutations and the other for validating the results. Candidate genes that might influence ICI treatment were identified by use of data from a cohort [MSKCC, Nat Genet 2019] which contained OS of ICI treatment [17]. We next validated the predictive power of 20-gene mutation signature from another cohort [MSKCC, J Clin Oncol 2018] [29] which contained PFS of ICI treatment and 86 patients from this cohort were sent for PD-L1 expression assessment. PFS and PD-L1 level were also included in another cohort [MSKCC, Cancer Cell 2018][30]. Mutations in these cohorts were derived by several various versions of the IMPACT targeted sequencing assay. Additional tumor mutation and survival data of NSCLC were obtained from the TCGA Pan-Cancer Atlas cohort [31].

### Mutation signatures and survival analysis

To evaluate if mutations in 20 potential genes could forecast the efficacy of the ICI treatment, we divided patients into three groups: those with no mutations in the 20 candidate genes (wild group), those with mutations in one or two of the 20 candidate genes (low mutation, or LM group), and those with three or more mutation in any of the 20 candidate genes (high mutation, or HM group).

The relationship between the gene mutation signature and other biomarker for immunotherapy.

Potential interactions between TMB or PD-L1 expression and the gene mutation signature in influencing efficacy of the ICI therapy were examined by a sub-cohort of these cohorts. Kaplan-Meier survival analysis was conducted and the patients were sorted by both their mutation signature and TMB or PD-L1 expression levels.

### CIBERSORT estimate of tumor‑infiltrating immune cell modulation

We performed CIBERSORT [32] analysis on RNA expression data from NSCLC patients to see if various mutation signatures correspond with particular features of the tumor microenvironment. The immune cell subset analysis of the tumor samples was performed using the publicly available online tool TIP [33], which is based on CIBERSORT principles.

### Development of nomogram

The Kaplan-Meier method was utilized to evaluate the prognosis univariates, and the log rank tests were employed to identify significant differences. A nomogram was created based on the outcomes of univariate analyses with the “rms” package of R software version 4.2.1. Concordance index (C-index) and calibration plot were used to estimate the accuracy and discriminative value of nomogram. This nomogram was verified by bootstrap analyses with 1000 resamples.

### Statistical analysis

Kaplan-Meier survival curves were constructed using the statistical program GraphPad Prism (version 8.2). Different patient groups were compared using log-rank test. All statistical analyses were performed using the IBM SPSS Statistics 25 and R software 4.2.1. All significant comparisons were considered as a two-tailed p-value < 0.05.

## Results

### Construction of 20-gene mutation signature

**Fig. 1:**
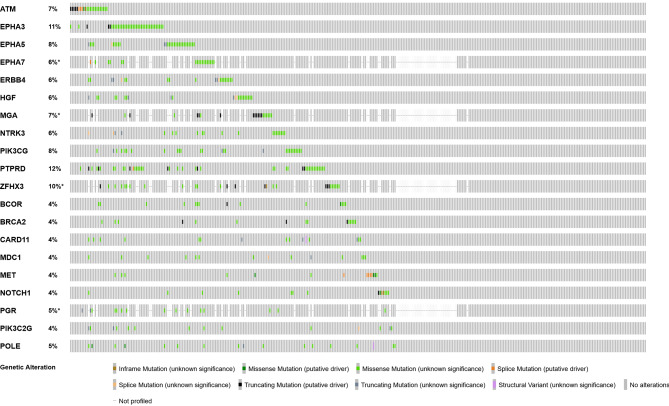
The prevalence of 20-selected gene mutations in a cohort [MSKCC, Nat Genet 2019] according to the cBioPortal for Cancer Genomics

Based on hypothesis that particular gene mutation functionally affected the efficacy of immunotherapy, we sought for genes whose mutations would improve the efficacy of ICI therapy by using the data from a cohort [MSKCC, Nat Genet 2019] which contained OS of ICI treatment (see Table S1 for patient baseline characteristics). Twenty potential genes were selected using the following standards:

(1) the gene must be mutated in at least ten of the patients; (2) The gene’s mutation frequency needs to be considerably greater in the surviving patients than it is in the deceased patients at the completion of the clinical observation period (with a *p* value 0.10 due to the small number of patients for some gene mutation). We solely focused on nonsysnomous alterations. Thus, gene fusions and amplifications were omitted. Applying these criteria to this cohort, top 20 mutated genes ranked by mutation frequency were selected. Mutation frequencies of 20 mutated genes which we analyzed are shown in Fig. 1.

**Fig. 2:**
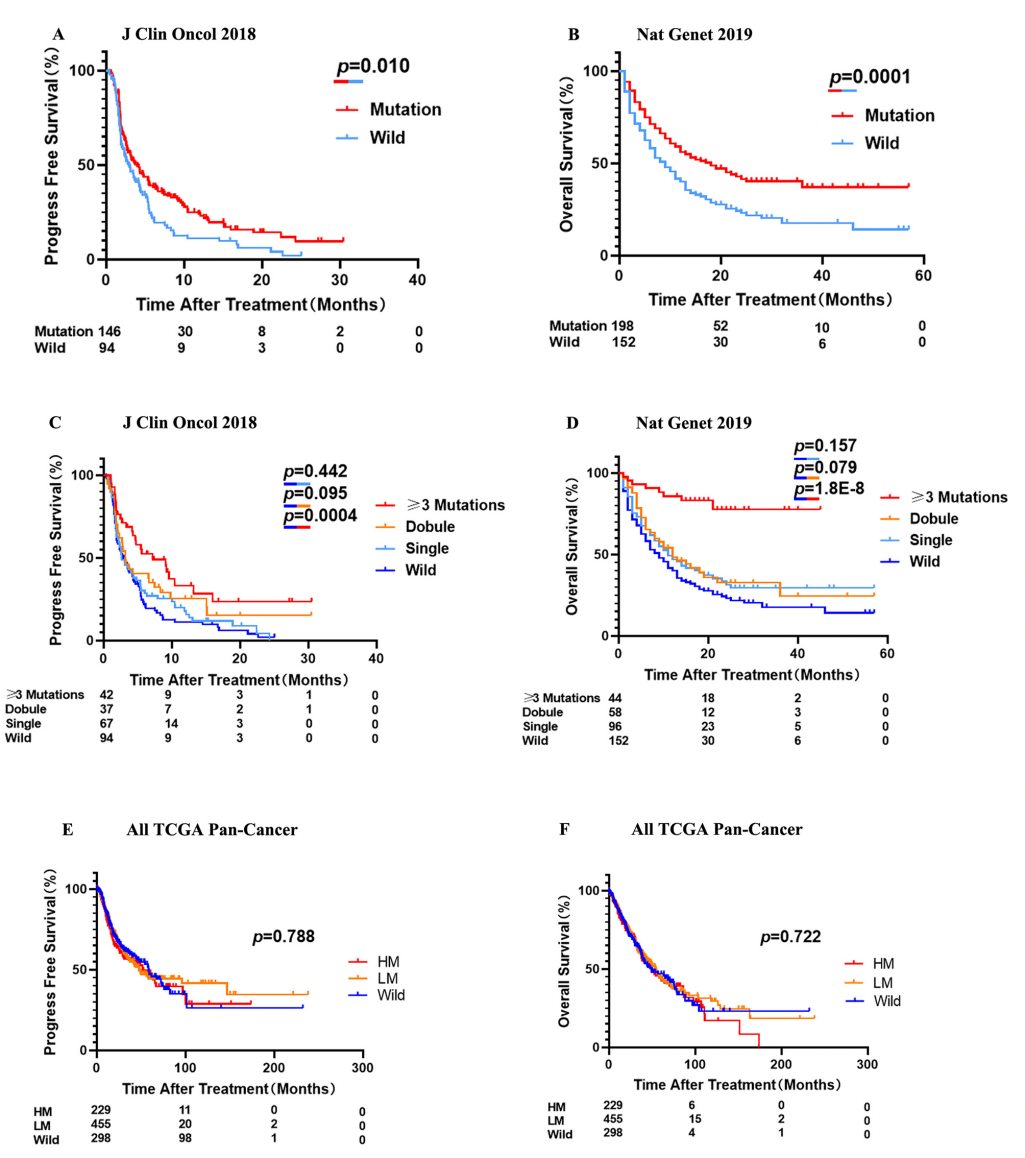
The high mutation signature in the 20-gene panel predicts for significantly better response to ICI treatment in NSCLC. **A.** PFS levels of the ICI treated NSCLC patients with 20-gene mutation and wild type. **B.** OS levels of the ICI treated NSCLC patients with 20-gene mutation and wild type. **C**. PFS levels in the ICI treated NSCLC patients with different 20-gene mutation signatures. **(D)** OS levels in the ICI treated NSCLC patients with different 20-gene mutation signatures. **(E)** PFS levels in the non-ICI treated NSCLC patients from the All TCGA Pan-Cancer cohort with different mutation signatures. **(F)** OS levels of the non-ICI treated NSCLC patients from the All TCGA Pan-Cancer cohort with different mutation signatures. P-values calculated by use of log-rank test

We next validated the predictive power of 20-gene mutation signature from another cohort [MSKCC, J Clin Oncol 2018] which contained PFS of ICI treatment (see Table S2 for patient baseline characteristics). The PFS and OS time period of the patients with mutation of 20 gene treated with ICI was longer than that of the wild type patients (the median PFS: 4.00 vs. 2.90 months, p = 0.010, HR = 0.70 (95% [CI]:0.52–0.93)); the median OS: 18 vs. 9 months, p = 0.0001, HR = 0.56 (95% [CI]:0.42–0.75), Fig. 2A, B). To find out which gene mutation characteristics could efficiently predict the efficacy of immunotherapy, NSCLC patients were divided into wild group, single mutation group, double mutation group, and ≥ 3 mutation group. Among them, OS and PFS in the single and double mutation groups were not significantly different compared with the wild group (PFS: Single group vs. Wild group: p = 0.442, Double group vs. Wild group: p = 0.095, Fig. 2C; OS: Single group vs. Wild group: p = 0.157, Double group vs. Wild group: p = 0.079, Fig. 2D). In contrast, OS and PFS of ≥ 3 mutation group were significantly improved compared with the wild group (the median PFS: 7.17 vs. 2.90 months, p = 0.0004, HR = 0.47 (95% [CI]:0.32–0.68), Fig. 2C; the median OS: unreached vs. 9 months, p = 1.8E-8, HR = 0.17 (95% [CI]:0.11–0.25), Fig. 2D). Because of the significant OS and PFS advantages of the ≥ 3 mutation group compared with other groups, we then divided the NSCLC patients receiving ICI treatment into three sub-cohorts with different mutation signature, including those without mutation in the 20 genes (Wild), those with mutations in just one or two of 20 genes (Low mutation, LM), and those with mutations in three or more of the 20 genes (High mutation, HM). In order to prove the observed clinical benefits in the high mutation group only existing in ICI treatment, not genetically inherent, a sub-cohort of NSCLC patients in the All TCGA Pan-Cancer cohort was analyzed. Our research revealed that the survival advantages shown in the high mutation group were not seen in the absence of ICI therapy (Fig. 2E, F). Therefore, we concluded that high mutation signature was a prognostic indicator for immunotherapy rather than prognostic for NSCLC in general.

### Comparation of predictive powers of 20-gene mutation signature with TMB level

**Fig. 3:**
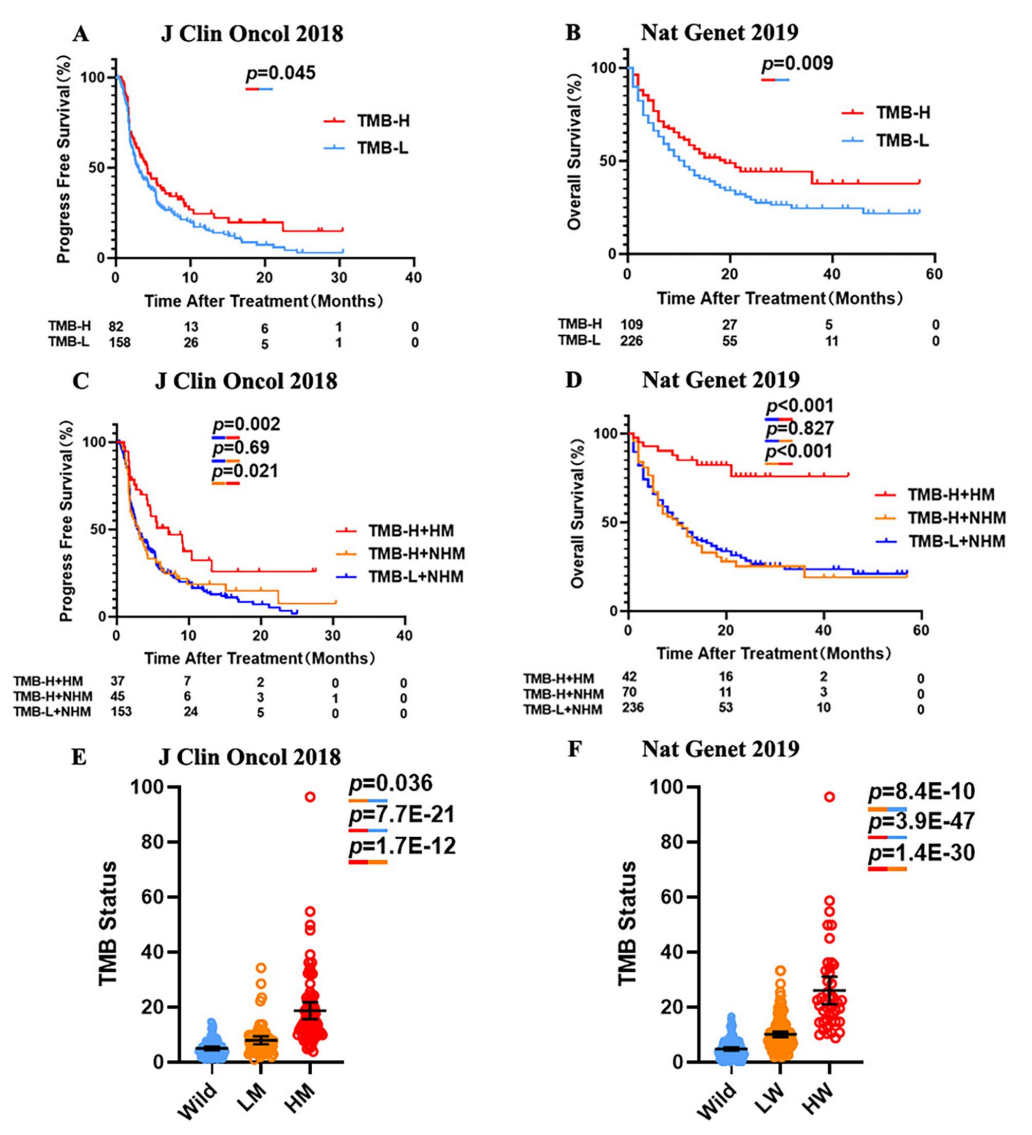
Comparation of predictive powers of 20-gene mutation signature with TMB level. **(A)** PFS levels of the NSCLC patients with high (≥ 10) and low (< 10) TMB values. **(B)** OS levels of the NSCLC patients with high (≥ 10) and low (< 10) TMB values. **(C)** PFS levels in the ICI treated NSCLC patients with TMB-H (≥ 10) and TMB-L (< 10) further stratified according to groups with high mutation signature (HM) or with no high mutation signature (NHM). **(D)** OS levels in the ICI treated NSCLC patients with TMB-H (≥ 10) and TMB-L (< 10) further stratified according to groups with high mutation signature (HM) or with no high mutation signature (NHM). **(E)** TMB levels in NSCLC patients [MSKCC, J Clin Oncol 2018] with different mutation signatures. **(F)** TMB levels in NSCLC patients [MSKCC, Nat Genet 2019] with different mutation signatures.P-values calculated by use of log-rank test (A,B,C,D) or unpaired t-test **(E, F)**

Recently, FDA approved TMB-H (≥ 10) as a biomarker of pembrolizumab treatment for solid tumors. We thus evaluated the predictive power of TMB in the both cohort of NSCLC patients. Patients with TMB-H demonstrated a significant survival advantage over those with TMB-L ((the median PFS: 4.20 vs. 3.03 months, p = 0.045, HR = 0.74 (95% [CI]:0.56–0.99); the median OS: 19 vs. 11 months, p = 0.009, HR = 0.58 (95% [CI]:0.43–0.79), Fig. 3A, B). Patients with high or low TMB were stratified by 20-gene mutation signature in order to assess the predictive value of TMB-H vs. 20-gene mutation signature levels (Fig. 3C, D). Because significant prognostic advantages of the ≥ 3 mutation in 20-gene mutation signature, we then defined ≥ 3 mutation in 20 genes as high mutation (HM) and others as non-high mutation (NHM). When compared PFS of NSCLC patients, only 5 patients were TMB-L with HM, so the log-rank test was made in other three groups. The results indicated that those patients with low TMB (< 10) and possessing the non-high mutation in 20-gene mutation signature had same PFS as those with high TMB and non-high mutation (p = 0.69, Fig. 3C). However, TMB-H with HM group (median PFS = 7.17 months) showed a significant survival advantage when compared with those with TMB-H + NHM (median PFS = 3.07 months, p = 0.021, HR = 0.55 (95% [CI]:0.33–0.91)) and those with TMB-L + NHM (median PFS = 3.03 months, p = 0.002, HR = 0.51 (95% [CI]:0.35–0.73)). 20-gene mutation signature showed the same predicted trend in OS of NSCLC patient. TMB-L + HM group only included two patients, and thus omitted in Kaplan-Meier survival curves. There is no difference of OS between TMB-L + NHM group and TMB-H + NHM group (p = 0.827, Fig. 3D). TMB-H + HM group (median OS unreached) showed a better prognostic compared with those with TMB-H + NHM (median OS = 10 months, p < 0.001, HR = 0.20 (95% [CI]:0.12–0.33)) and those with TMB-L + NHM (median OS = 10 months, p < 0.001, HR = 0.21 (95% [CI]:0.14–0.31)). In both cohorts, a higher TMB value was confirmed in HM group, compared with LM and Wild groups (LM group: p = 7.7E-21, Wild group: p = 1.7E-12, Fig. 3E; LM group: p = 3.9E-47, Wild group: p = 1.4E-30, Fig. 3F). Therefore, our analysis clearly demonstrated that nearly all patients with high mutation in 20 genes belong to those with TMB-H. Moreover, among patients with TMB-H, only those with high mutation could benefit from ICI treatment. This result could be applied to exclude patients with TMB-H but no ICI benefit. These results indicated that the superiority of 20-gene mutation signature in predicting the efficacy of ICI treatment compared with TMB (≥ 10) in NSCLC patients.

Independence of the 20-gene mutation signature from PD-L1 in predicting ICI treatment response.

**Fig. 4:**
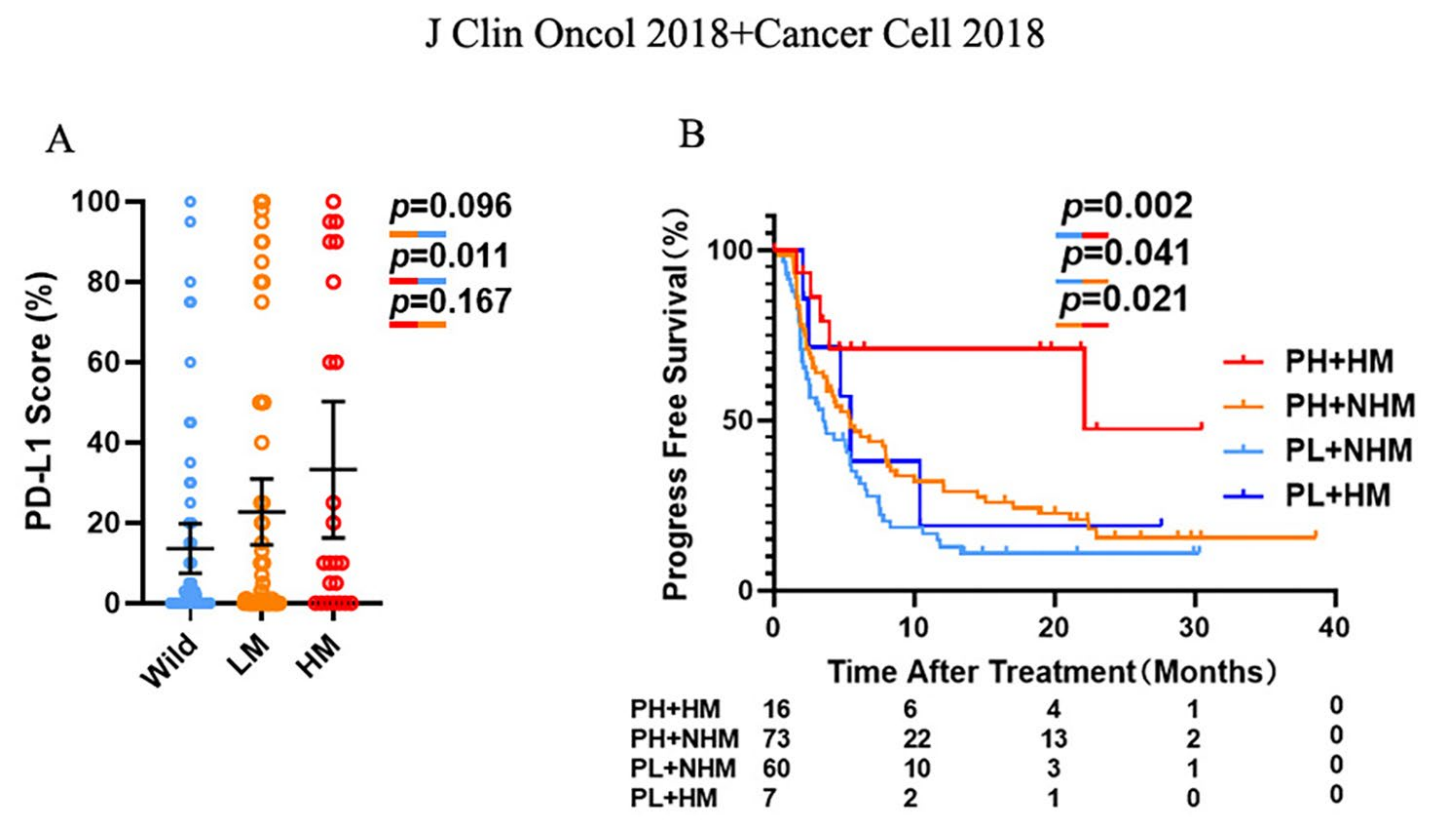
Independence of the 20-gene mutation signature from PD-L1 in predicting ICI treatment response. **(A)** PD-L1 expression levels in NSCLC patients with different mutation signatures. **(B)** PFS levels of patients stratified according to their PD-L1 expression levels and mutation signature status. PL-NHM: PD-L1 level low (< 1%), no high mutation signature; PH-NHM: PD-L1 high (≥ 1%), no high mutation signature; PL-HM: PD-L1 low (< 1%), high mutation signature; PH-HM, PD-L1 high (≥ 1%), high mutation signature. P-values calculated by use of unpaired t-test **(A)** or log-rank test **(B)**

Because PD-L1 was currently the most widely used biomarker in immunotherapy, we then evaluated the relationship between the 20-gene mutation signature and PD-L1 levels in predicting the efficacy of ICI treatment. This study analyzed the cohorts of NSCLC patients from MSKCC for whom the PD-L1 expression data were accessible. Patients with all three mutation signatures had varying amounts of PD-L1 expression, but high mutation group was not statistically different from low mutation groups (p = 0.167, Fig. 4A). Importantly, survival analysis revealed that among cohorts of high PD-L1 expression, the high mutation group had a considerably superior PFS rate, compared with non-high mutation group (p = 0.021, HR = 0.36 (95% [CI]:0.19–0.67), Fig. 4B). As a result, the high mutation signature could predict ICI benefits regardless of PD-L1 expression levels.

Establishment of a prognostic nomogram for the prognosis of ICI treatment in NSCLC.

**Fig. 5:**
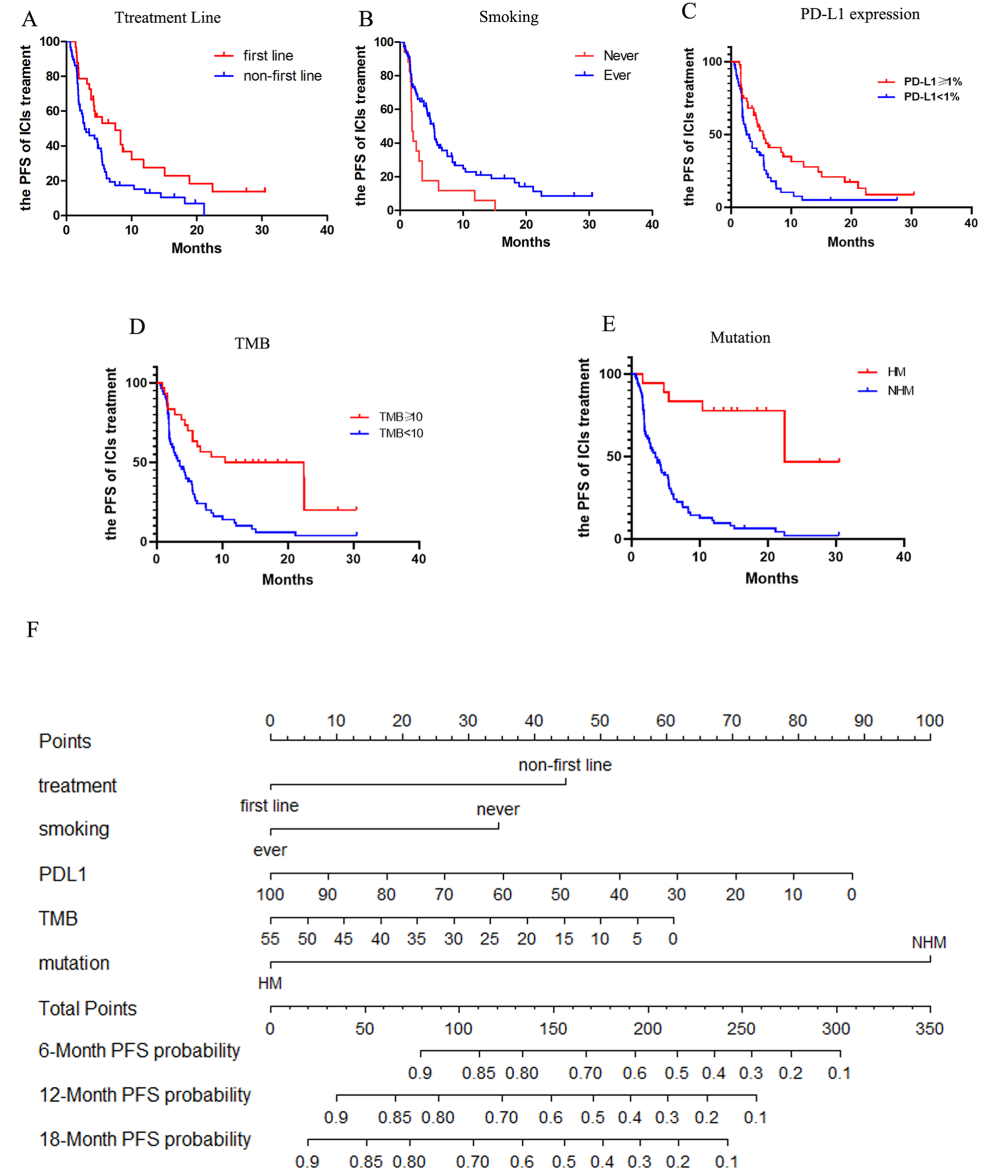
Establishment of a prognostic nomogram for the prognosis of ICI treatment in NSCLC. **(A)** The survival curves for ICIs-treated patients based on treatment lines; **(B)** The survival curves for ICIs-treated patients based on smoking history; **(C)** The survival curves for ICIs-treated patients based on PD-L1 expression; **(D)** The survival curves for ICIs-treated patients based on the tumor mutational burden (TMB); **(E)** The survival curves for ICIs-treated patients based on 20-gene mutation signature. **(F)** The novel nomogram based on patient information to predict the prognosis of ICI treatment. P-values calculated by use of log-rank test

**Fig. 6:**
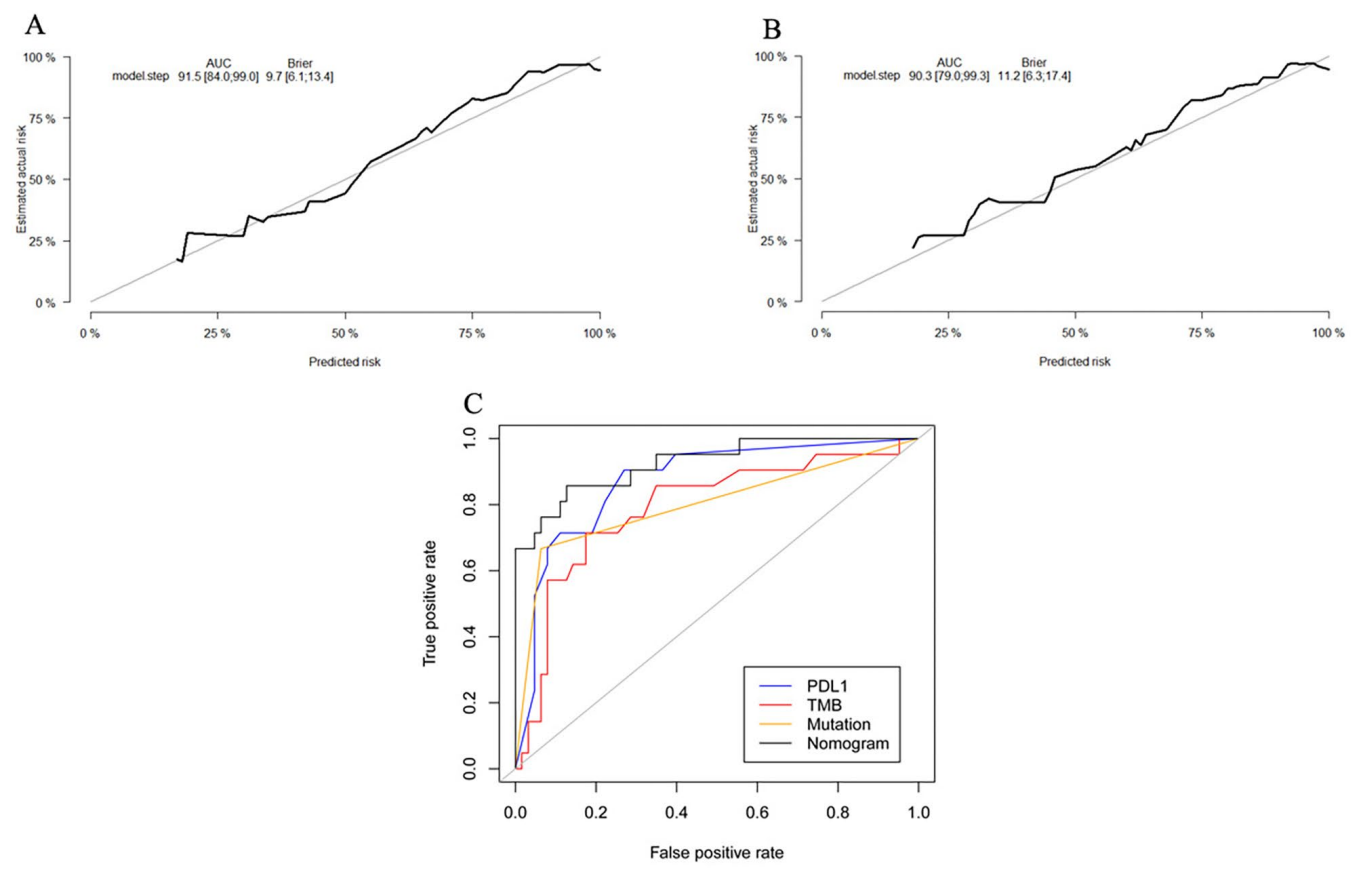
Evaluation of a prognostic nomogram for the prognosis of ICI treatment in NSCLC. **(A)** The calibration plot for the nomogram. **(B)** Internal validation of the nomogram using the bootstrap sampling. **(C)** ROC curves were used to compare different prognosis indicators

We constructed a nomogram to predict the PFS of 86 NSCLC patients of the selected cohort [MSKCC, J Clin Oncol 2018] based on the integrated information of clinicopathologic features, targeted sequencing, and PD-L1 expression. Firstly, the univariate analyses were performed in the identification of the variables for nomogram construction. Multiple variables were proved to be significantly associated with the PFS of NSCLC patients with ICI treatment, including treatment lines(p = 0.0097), smoking(p = 0.0040), PD-L1 expression(p = 0.0112), TMB(p = 0.0380) and 20-gene mutation signature(p = 0.0030) (Fig. 5A-E). Furthermore, the univariate analysis revealed that advanced NSCLC patients with high mutation signature, ever smoking, first-line ICI treatment, higher PD-L1 expression (≥ 1% percentage) or a high TMB score (≥ 10), could benefit from immunotherapy. The nomogram based on these variables was then established, as shown in Fig. 5F. This prognostic model’s C-index was 0.733(95%CI 0.700–0.766), which indicated that it has a relatively robust ability to predict the PFS of advanced NSCLC patients treated with ICI. Calibration plots (Fig. 6A) demonstrated good diagnostic performance in predicting the PFS of 12 months. Furthermore, the model’s area under the ROC curve (AUC) was 0.915(95%CI 0.840–0.990) and the brier index was 9.7(95%CI 6.1–13.4). Internal bootstrap validation was used to validate the nomogram for predicting 12-month PFS. The calibration plot was measured by bootstrapping for 500 repetitions, and the AUC of the bootstrap stepwise model was 0.903(95%CI 0.790–0.993) and brier index was 11.2(95%CI 6.3–17.4), with a statistical power similar to that of the initial stepwise model (Fig. 6B). ROC curve analysis for the 12-month PFS showed that nomogram (AUC = 0.915(95%CI 0.840–0.990)) had better predictive ability than single biomarker (PD-L1: AUC = 0.872(95%CI 0.875–0.959); TMB: AUC = 0.792 (95%CI 0.673–0.910); Mutation: AUC = 0.802 (95%CI 0.674–0.930)). (Fig. 6C)

### The role of 20-gene mutation signature in tumor‑infiltrating immune cell (TIIC) modulation

**Fig. 7:**
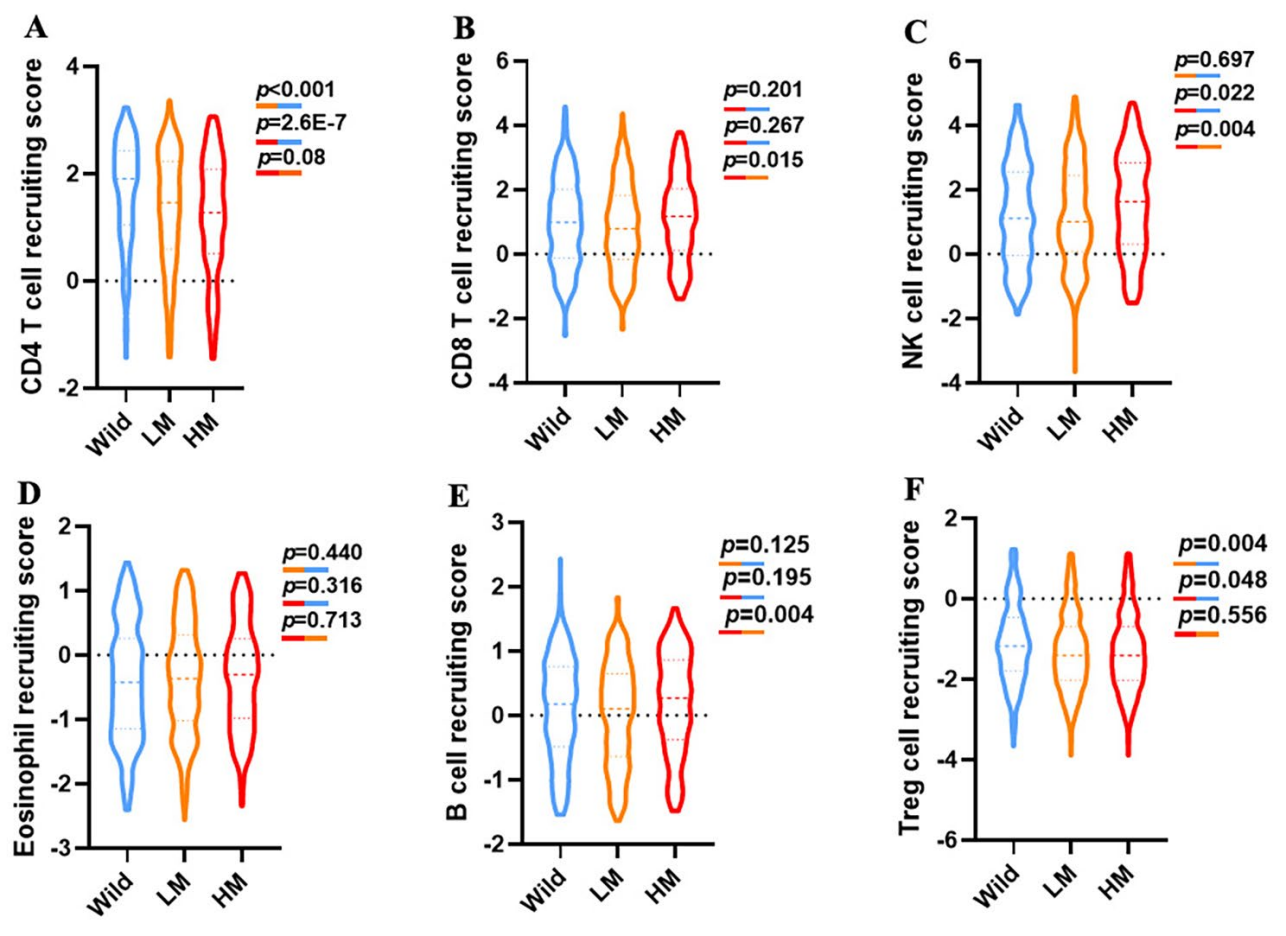
The role of 20-gene mutation signature in tumor‑infiltrating immune cell modulation. A cohort of NSCLC patients from the TCGA Pan-Cancer Atlas were analyzed for their CIBERSORT immune effector scores based on their mutation signatures. **(A)** Recruitment scores for CD4 T cells; **(B)** Recruitment scores for CD8 cells; **(C)** Recruitment scores for NK cells; **(D)** Recruitment scores for eosinophils; **(E)** Recruitment scores for B cells; **(F)** Recruitment scores for Treg cells; P-values calculated by use of t-test (unpaired, two-tailed)

We hypothesized that 20-gene mutation might influence the biology of the malignancies directly, besides the provision of neoantigens recognized by the immune system. As a result, 20-gene mutation signature might influence tumor‑infiltrating immune microenvironment. To verify this theory, we used the online program tool TIP40 to evaluate gene expression data from 501 NSCLC patients (TCGA pan cancer atlas cohort) based on their mutation signatures using the CIBERSORT algorithm. CIBERSORT algorithm could accurately identify various immunoeffector cellular subgroups in tumor tissues. Our study revealed that tumors with the high mutation signature exhibited considerably more intratumoral infiltration of CD8 T cells (Fig. 7B), NK cells (Fig. 7C), and B cells (Fig. 7E) in the NSCLC patients from TCGA pan cancer atlas cohort. However, intratumoral infiltration of CD4 T cells (Fig. 7A) and Treg cells (Fig. 7F) in the high mutation group were significantly reduced. There was no difference of intratumoral infiltration of Eosinophil between three subgroups (Fig. 7D). These findings implied that the 20-gene mutation signature could predict a pro-inflammatory tumor immunological microenvironment in NSCLC.

## Discussion

Treatment of NSCLC has been revolutionized by ICI treatment. However, only a subset of patients could benefit from ICI treatment. Nowadays, mutation panels have been used as novel predictive markers for ICI treatment. Xavier et al. firstly proposed that combining TMB and mutation profiles in key cancer genes could better qualify patients for ICI treatment and predict their OS [34]. However, this study only showed that these genes were associated with prognosis of patients for ICI treatment, and did not construct them into a complex mutation panel. On the basis of machine learning, Peng et al. exploited a somatic mutation signature to predict the best overall response to PD-1 therapy in NSCLC [35]. The AUC of somatic mutation signature in the cohorts were higher than those of TMB and PD-L1 expression. This study focused on constructing a prediction model for PD-1 therapy, but lacked a subgroup analysis of gene mutation signature and TMB/PD-L1 expression, which could compare the prediction advantages of the mutation panel with TMB/PD-L1 expression. Pan et al. firstly constructed a complex gene mutation signature, using the standard of a significant difference in mutation frequencies between surviving and deceased patients [36]. Moreover, subgroup analysis showed that mutations in select genes might be a better predictor of NSCLC response to ICI therapy than TMB-H. The shortcoming of this gene mutation panel was that it contained 52 genes, and most genes had low mutation frequencies Therefore, this gene panel might be not suitable for clinical application. In conclusion, our gene panel had the advantage of a small number of genes and high mutation frequencies. Moreover, it could screen for poor efficacy in patients with TMB-H and better efficacy in PD-L1 positive expressers.

At present, PD-L1 expression was regarded as the most used biomarker for ICI treatment of NSCLC patients. However, it is a challenge for advanced NSCLC patients to assess the expression of PD-L1, given difficulties in tissue sample acquisition, preservation and its predicted uncertainty. In contrast, the identification of 20-gene mutation could be achieved with various platform technologies, such as archival FFPE tissues and liquid biopsies including blood or saliva samples. Therefore, it seemed easier for the implement of 20-gene mutation signature as a novel biomarker in the clinic. Moreover, we found that among cohorts with high PD-L1 expression, the high mutation group had a considerably superior prognosis, compared with non-high mutation group. However, its utility in the clinic, especially when compared with TMB or PD-L1, should be assessed in upcoming prospective clinical trials.

Our 20-gene mutation signature was chosen using the standard of a significant difference in mutation frequencies between surviving and deceased patients, rather than their specific biological functions. We only included genes with top 20 of mutation frequency in order to ensure that these mutations were easily to be found in the clinic. This was necessary for a novel indicator of ICI treatment to be widespread in the future. Through different combination of mutated genes, ≥ 3 gene mutations were affirmed as a clear cut-off to predict the efficacy of ICI treatment. It is worth looking forward to that predictive power of this biomarker would been improved by enhancing the quality of various gene panel. Besides, a novel nomogram was constructed based on 20-gene mutation signature, PD-L1 expression, TMB level, and other clinical features of advanced NSCLC patients with ICI therapy based on the Cox regression model established in this research. ROC curve analysis showed that nomogram had better predictive power in the efficacy of ICI treatment than other biomarkers. This might assist physicians to estimate the clinical benefits of immunotherapy and develop the appropriate therapeutic proposal and follow-ups before advanced NSCLC patients received therapies.

Published literature indicated that ATM and BRCA2 were vital genes in regulating DNA double strand break repair. These genes were linked to benefiting from immunotherapy and made an important role in cellular innate immunity [37, 38]. In addition, POLE was involved in DNA replication and had a crucial role in DNA synthesis and repair [39, 40]. Furthermore, tumors with POLE-mutated showed a high expression in effector cytokines, T lymphocytes markers and a significant up-regulation of immune checkpoints genes, such as PD-1, PD-L1 and CTLA4 [41]. Expression of NTRK3 was previously positively associated with higher stromal scores, improved immune response and a great variety of immune lymphocytes [42]. The PTPRD gene could up-regulate mRNA expression of JAK1 and STAT1, which promoted expression of chemokines to attract T cells [43]. EPHA3, as a type of transmembrane receptor, was identified as a binding partner for PD-L1, and EphA3/PD-L1 co-expression was associated with a CD8 + effector cell signature [44]. On the other hand, any potential mechanism of how some other genes in 20-gene panel were involved in tumor response to immunotherapy was unknown. Therefore, they were suitable for testing in wet lab studies.

It was well-known that the type of tumor immune microenvironment (TIME) plays an important role in cancer cell elimination [45, 46], with “hot” TIME facilitating cancer cell killing. According to our findings, the 20-gene mutation signature was strongly associated with multiple immune cell infiltration. Moreover, positive correlations were observed between high mutation signature and immunoeffector cellular subgroups (CD8 T cell, NK cell, B cell). We speculated that it might be that the high mutation signature promotes both a reasonable high TMB and the formation of “hot” TIME.

At present, lots of NSCLC patients only sent for PD-L1 expression and TMB assessment, and some of them do not benefit from immunotherapy. In fact, such simple evaluation is insufficient. This study proves the importance of genetic testing before immunotherapy. For those patients with PD-L1 positive expression or TMB-H, it is recommended to assess 20-gene mutation signature. We found that TMB-H patients without high mutation signature could not benefit from ICI therapy, which was a supplement to FDA’s criteria for TMB-H. Nevertheless, several limitations of the study herein have been presented as follows. Development and validation were based on cohorts from retrospective studies, which might diminish the validity of the claimed conclusions. In the future, it was a necessary step to validate these claims through randomized clinical trials before clinical application.

## Conclusion

Our study indicated that a high mutational signature with 3 or more of the 20-gene panel could provide more accurate predictions for the outcomes of ICI therapy than TMB ≥ 10 in several published cohorts. Before it can be used as a predictive biomarker for selecting patients who can benefit from ICI treatment, further clinical trials needed to be conducted to provide adequate evidence.

## Electronic supplementary material

Below is the link to the electronic supplementary material.

Supplementary Material 1

## Data Availability

All data generated during this study are included in this published article. The datasets generated in the current study are available in the cBioportal for Cancer Genomics (http://www.cbioportal.org/).

## Abbreviations

NSCLC: Non-small cell lung cancer
MMR: Mismatch repair
MSI-H: Microsatellite instability-high
PD-L1: Programmed death ligand 1
TMB: Tumor mutational burden
ICIs: Immune checkpoint inhibitors
TIICs: Tumor-infiltrating immune cells
TIME: Tumor immune microenvironment
TCGA: The Cancer Genome Atlas
PFS: Progress free survival
MSKCC: Memorial Sloan-Kettering Cancer Center
