# Supplementary material for: A 20-gene mutation signature predicts the efficacy of immune checkpoint inhibitor therapy in advanced non-small cell lung cancer patients
Source: BMC Pulm Med. 2023 Jun 22;23:223. doi: 10.1186/s12890-023-02512-6 (PMC10288780; doi:10.1186/s12890-023-02512-6)
Supplement: Supplementary file 1 — Supplementary Material 1 [file 12890_2023_2512_MOESM1_ESM.docx]

| NSCLC patient characteristics | Wild（N=152） | LW（N=154） | HW（N=44） | p value |
| --- | --- | --- | --- | --- |
| Age (mean (SD)) | 64.75 (11.90) | 67.36 (9.86) | 63.23 (8.58) | 0.027 |
| Sex, n (%) | | | | |
| Male | 71(46.7%) | 78(50.6%) | 21(47.7%) | 0.783 |
| Female | 81(53.3%) | 76(49.4%) | 23(52.3%) |  |
| Tumor type (%) | | | | |
| Lung Adenocarcinoma, n (%) | 122 (80.3%) | 114 (74.0%) | 35 (79.5%) | 0.823 |
| Lung Squamous Cell Carcinoma, n (%) | 19 (12.5%) | 22 (14.3%) | 4 ( 9.1%) |  |
| Poorly Differentiated Non-Small Cell Lung Cancer, | 4 ( 2.6%) | 7 ( 4.5%) | 2 ( 4.5%) |  |
| other, n (%) | 7 ( 4.6%) | 11 ( 7.1%) | 3 ( 6.8%) |  |
| Sample Type | | | | |
| Primary, n (%) | 75(49.3%) | 75(48.7%) | 21(47.7%) | 0.981 |
| Metastasis, n (%) | 87(50.7%) | 79(51.3%) | 23(52.3%) |  |
| Overall Survival Status | | | | |
| Living, n (%) | 41(27%) | 55(35.7%) | 35(79.5%) | <0.001 |
| Deceased, n (%) | 111(73%) | 99(64.3%) | 9(20.5%) |  |
| TMB score (mean (SD)) | 4.83 (3.38) | 10.13 (5.87) | 26.07 (16.50) | <0.001 |
| Drug Type | | | | |
| PD-1/PDL-1, n (%) | 143(94.1%) | 145(94.2%) | 41(93.2%) | 0.97 |
| PD-1/PDL-1 &CTLA4, n (%) | 9 (5.9%) | 9 (5.8%) | 3(6.8%) |  |

Table S1. Patient characteristics in the cohort[MSKCC, Nat Genet 2019]
*P values were calculated by use of unpaired t-test. Other P values were calculated by use of Chi-Squared Test.

Table S2. Patient characteristics in the cohort[MSKCC, J Clin Oncol 2018]

| NSCLC patient characteristics | Wild（N=94） | LW（N=104） | HW（N=42） | p value |
| --- | --- | --- | --- | --- |
| Age (mean (SD)) | 63.73 (13.91) | 66.01 (9.70) | 62.14 (8.94) | 0.136 |
| Sex, n (%) | | | | |
| Male | 50(53.2%) | 51(49.0%) | 17(40.5%) | 0.391 |
| Female | 44(46.8%) | 53(51.0%) | 25(59.5%) |  |
| Tumor type (%) | | | | |
| Lung Adenocarcinoma, n (%) | 74 (78.7%) | 81 (77.9%) | 31 (73.8%) | 0.926 |
| Lung Squamous Cell Carcinoma, n (%) | 13 (13.8%) | 15 (14.4%) | 6 (14.3%) |  |
| other, n (%) | 7 (7.4%) | 8 (7.7%) | 5 (11.9%) |  |
| Smoking History | | | | |
| Ever, n (%) | 62 (66.0%) | 89 (85.6%) | 42(100%) | <0.001 |
| Never, n (%) | 32(34.0%) | 15(14.4%) | 0(0%) |  |
| Progression Free Status | | | | |
| Progressed, n (%) | 84(89.4%) | 88(84.6%) | 26(61.9) | <0.001 |
| Not Progressed, n (%) | 10(10.6%) | 16(15.4%) | 16(38.1) |  |
| TMB score (mean (SD)) | 5.01 (3.18) | 9.77 (6.32) | 23.62 (16.58) | <0.001 |
| Drug Type | | | | |
| PD-1/PDL-1, n (%) | 77(81.9%) | 94(90.4%) | 35(83.3%) | 0.204 |
| PD-1/PDL-1 &CTLA4, n (%) | 17(18.1%) | 10(9.6%) | 7(16.7%) |  |
| Durable Clinical Benefit | | | | |
| Yes, n(%) | 17(19.3%) | 34(33.3%) | 18(48.6%) | 0.003 |
| No, n(%) | 71(80.7%) | 68(66.7%) | 19(51.4%) |  |
| PD-L1 Score (mean (SD)) | 13.38 (26.85) | 25.07 (35.13) | 36.54 (43.70) | 0.096 |

*P values were calculated by use of unpaired t-test. Other P values were calculated by use of Chi-Squared Test.
